# Eosinophilic meningitis caused by *Angiostrongylus cantonensis:* case report in a patient with false-positive immunological test result for *Neisseria meningitides*

**DOI:** 10.1590/S1678-9946202668017

**Published:** 2026-02-16

**Authors:** Marcela Nataly Parra Alvarez, Juan Sebastián Sánchez León, Tassiane Moreira da Silva, Renato Dumba Monteiro de Castro, Arlete Hilbig, Leyva Cecilia Vieira de Melo, Carlos Graeff-Teixeira, Alessandro Comarú Pasqualotto

**Affiliations:** 1 Santa Casa de Porto Alegre, Porto Alegre, Rio Grande do Sul, Brazil; 2 Universidade Federal de Ciências da Saúde de Porto Alegre, Porto Alegre, Rio Grande do Sul, Brazil; 3 Instituto Adolfo Lutz, São Paulo, São Paulo, Brazil; 4 Universidade Federal do Espírito Santo, Vitória, Espírito Santo, Brazil

**Keywords:** Eosinophilic meningitis, Angiostrongylus cantonensis, Food-borne diseases

## Abstract

Eosinophilic meningitis caused by *Angiostrongylus cantonensis* is an uncommon disease in Brazil that occurs by ingesting slugs, water or food contaminated with the parasite. Here we report a case of a 64-year-old patient with neck stiffness, headache, fever, and peripheral and cerebrospinal fluid (CSF) eosinophilia, compatible with eosinophilic meningitis. He had a false-positive result for *Neisseria meningitides* and received unsuccessful treatment with antibiotics. After extensive investigation, DNA and anti*-A.cantonensis* antibodies were detected and the patient was successfully treated with prednisone and albendazole. Diagnosis of neuroangiostrongyliasis relies on epidemiological data, as well as clinical and laboratory examinations such as detection of antibodies and DNA of the parasite in CSF. Its timely treatment with corticosteroid therapy reduces damage to neural tissues and manages headache.

## INTRODUCTION

Meningitis may be lethal if not properly treated. Eosinophilic meningitis is characterized by the presence of >10 eosinophils/mm^3^ in the cerebrospinal fluid (CSF) or the equivalent of >10% of the total leukocytes in the CSF^1,3^. The etiological agents for this type of disease are usually parasites, most commonly *Angiostrongylus cantonensis* ^1^.

First described in Taiwan^1^, the disease may have spread worldwide due to maritime trade. In Brazil, the first reported cases occurred in the Espirito Santo State (Cariacica and Vila Velha) and in the Pernambuco State (Recife)^2^. Many other cases have been reported in states like Sao Paulo and Rio Grande do Sul. Although classified as a notifiable disease due to its potential severity, until 2020 only 35 cases were confirmed and 84 were reported as suspected cases in Brazil, reason why it is considered rare^2^. Meningitis is a disease requiring mandatory notification within 24 h and outbreaks, clustered cases, and deaths must be reported immediately. All suspected or confirmed cases must be reported to surveillance authorities by healthcare professionals and public and private laboratories, filling out the SINAN (Complaint Reporting Information System) form.

Clinical manifestations usually involve neck stiffness, headache, nausea and, sometimes, vomiting^3^. Risk factors involve ingestion of snails, slugs, raw shrimp, or other types of food, like poorly washed vegetables and fruits with the mucus of mollusks^4^.

*Angiostrongylus cantonensis* lives in the pulmonary arteries of rats, which is considered the definitive host while mollusks are considered intermediate guests where it is found in the larval stage that develops in the fibromuscular tissue of these animals^4,5^. Humans may ingest contaminated mollusks or vegetables, or even contaminated water (main method of transmission), and become accidental hosts. The larvae lodge in the human central nervous system and begin to produce inflammation of the meninges, as well as the aforementioned neurological symptoms^6^.

### Ethics

Written informed consent was obtained from the patient.

## CASE REPORT

**Figure 1:**
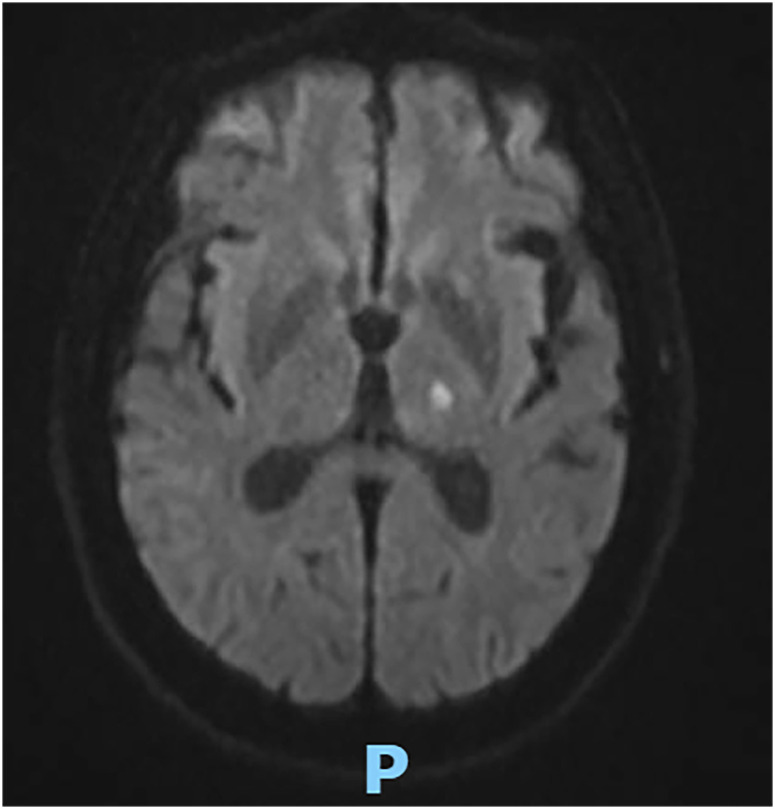
Magnetic resonance of the skull. Focus on diffusion restriction in the left thalamus compatible with an acute ischemic event.

We report the case of a 64-year-old man from Viamao (30° 04′ 51″ South, 51° 01′ 22″ West, Rio Grande do Sul State, Brazil), alcoholic and previously healthy. The patient headed to the emergency service due to a neurological condition characterized by confusion, drowsiness, speech aphasia, and motor deficit in the lower limbs that had lasted 5 days. Upon arrival, the patient was in good general condition: stable vital signs, no fever, but with mild dysarthria and stiffness of the cervical muscles and a positive Brudzinski sign. Additional tests revealed mild blood leukocytosis (13,140 cells/mm^3^) with eosinophilia (1,275 cells/mm^3^). Magnetic resonance of the skull showed signs of minimal supratentorial microangiopathy with focal hypodensity in the left thalamus, probably related to a recent ischemic event (Figure 1). Cerebrospinal fluid (CSF) analysis revealed an increased protein concentration (201 mg/dL) and altered cellularity (714 leukocytes/mm^3^, with 73% eosinophils and CSF glucose of 35 mg/dL). The antigen test (kit latex pastorex meningitis Bio-Rad) was positive for *Neisseria meningitidis* group B, and treatment was initiated with ceftriaxone 2 g for 14 days and a 5 day course of corticosteroid therapy although CSF was not suggestive of bacterial meningitis. The patient initially showed a decrease in leukocytosis and peripheral eosinophilia, but without a significant improvement in his neurological condition.

A new CSF sample was collected on the 7th day of antibiotic treatment, showing slight improvement in biochemical parameters, with protein concentration of 171 mg/dL and persistent abnormal cellularity (354 leukocytes/mm^3^, with 33% eosinophils and CSF glucose of 36 mg/dL). CSF bacterial culture, cryptococcal antigen test, VDRL, *Strongyloides stercoralis* test, herpes simplex types 1 and 2 PCR were negative. Serological tests for HIV, HCV, HBsAg, syphilis, Chagas disease and toxoplasmosis were negative. Parasitological examinations of stool were also negative, as was the research on *S. stercoralis* larvae by the Baermann-Moraes method.

After 14 days of antibiotic therapy without clinical improvement in neurological symptoms, an alternative etiological diagnosis was considered. Cerebrospinal fluid (CSF) and serum samples were submitted to the Adolfo Lutz Institute (Sao Paulo, Brazil). Anti–*Angiostrongylus cantonensis* IgG antibodies were detected in both serum and CSF by enzyme immunoassay using crude antigen from the *A. cantonensis* Village strain. Additionally, *A. cantonensis* DNA was detected in CSF by polymerase chain reaction (PCR) using the primers CO1ACF7 (5′-TGC CTG CTT TTG GGA TTG TTA GAC-3′) and CO1ACR7 (5′-TCA CTC CCG TAG GAA CCG CA-3′)^7^. IgG detection by Western Blot analysis was positive in the CSF and indeterminate in the blood, considering the recognition of the 29, 30, and 31 kDa fractions^8,9^.

The patient received targeted therapy for confirmed *Angiostrongylus cantonensis* infection with a 15-day regimen of oral albendazole (400 mg/day) and prednisone (60 mg/day, with subsequent progressive weaning). Following treatment initiation, substantial neurological improvement was observed. Clinical outcomes included a recovery of the patient's state of consciousness and orientation parameters, as well as significant improvement in dysarthria.

Upon secondary review of the patient's epidemiological history, consumption of raw foods was explicitly denied. Notably, the patient reported a high abundance of potential intermediate hosts (slugs) within their environment. Prior to hospital discharge, a control collection of cerebrospinal fluid (CSF) was performed. Analysis indicated favorable trends in cellularity and protein concentration parameters, with eosinophil counts approaching the normal reference range. All diagnostic data and test results acquired during the hospitalization period are summarized comprehensively in Table 1.

**Table 1:** Result of cerebrospinal fluid and serum analyzes during hospitalization in a patient with eosinophilic meningitis due to *Angiostrongylus cantonensis.*

	Cerebrospinal fluid							Serum		
Day	Glucose (mg/dL)	Protein (mg/dL)	Lactate (mmol/L)	Erythrocytes (µL)	Leukocytes (µL)	Differential (%)	Diagnostic tests	Leukocytes (µL)	Eosinophils (µL)	C reactive protein (mg/L)
1	35	201	3.0	1	714	E:73% L:19%	Negative microscopy Negative culture for bacteria Negative AFB Negative TB PCR Negative mycological tests Negative CrAg Herpes 1-2 negative Syphilis negative *S. stercoralis* negative **Positive *N.meningitidis* Group B antigen test**	13,140	1,275	136
7	36	171	2.4	1,925	354	E:33% L:63%		7,600	547	208
15	34	131	2.3	3	320	E:18% L:79%	**ELISA IgG positive for *A. cantonensis*** **Western blot positive for *A. cantonensis*** ***A.cantonensis* DNA detected**	5,010	331	182
35	52	77	2.3	503	305	E: 10% L: 85%		9,240	142	11

AFB = acid-fast bacillus; CrAg = *Cryptococcus* antigen; E = eosinophils; ELISA = enzyme immunoassay; L = leukocytes; PCR = polymerase chain reaction; TB = tuberculosis.

## DISCUSSION

Eosinophilic meningitis is a rare and potentially deadly disease requiring clinical suspicion at early stages. A study that analyzed symptoms in 484 people with eosinophilic meningitis showed that 99% suffered from headache and more than half (64%) presented neck stiffness, while fever occurred in 37%^9^. Atypical manifestations include seizures, facial paralysis, and drowsiness^9^. These patients usually present with peripheral eosinophilia, a finding associated with a sensitivity of 77% and specificity of 80%^9^. Imaging investigation may be common in 55% of cases^9^. Abnormal findings on cranial nuclear magnetic resonance include meningeal and globus pallidus enhancement. Another study described microhemorrhages and multiple microcavities which may be related to the movement of larvae through the central nervous system^9^.

CSF in patients with eosinophilic meningitis may appear clear or sometimes cloudy. In addition to eosinophilia, other immunological methods for detecting meningitis caused by *A. cantonensis* use purified antigens or crude extracts, but the 31 kDa antigen showed 100% sensitivity and specificity. However, one study found cross-reactivity with trichuriasis, trichinellosis, and opisthorchiasis when 31 kDa was used in Western Blotting, as well as cross-reactivity with *Toxocara canis* with some purified antigens^9^. Cross-reaction of *A.cantonensis* and bacterial antigens has never been reported. Latex agglutination test was initially positive for *Neisseria meningitidis* in the studied patient, and cross-reactions with rheumatoid factor and other bacterial antigens are known to occur^10^. False positive results could be explained by the possibility of cross-reactivity in the case of antigenic similarity between capsular polysaccharides from different bacterial species; therefore, there is identity between the K1 antigen from *E. coli* and a polysaccharide from *N. meningitidis* group B^11^. Cases of false positive for *N. meningitidis* group B in the CSF have been described in the literature, but the test has a sensitivity of 80% and a specificity of 97%^10^. Meningitis can be caused by more than two microorganisms at the same time. Some studies report cases of cryptococcal, tuberculous, and streptococcal meningitis occurring simultaneously^12^. This could have occurred in our case study; however, the characteristics of the cerebrospinal fluid were not compatible with bacterial meningitis. Moreover, PCR for detecting *Neisseria meningitidis* DNA was not considered since antigen tests have high sensitivity and specificity for diagnosing meningococcal meningitis.

The American Society of Infectious Diseases recommends initial CSF analysis, along with Gram and bacterial culture. If the Gram is negative, the latex agglutination test is recommended to identify the most common pathogens and finally, if available, PCR should be performed for viruses and bacteria^13^.

Among molecular tests, PCR can be identified early in samples from patients infected with *A. cantonensis*. In the presented case, anti*-Angiostrongylus* IgG were identified against the crude antigen of *A. cantonensis,* Village strain, in bands of 29, 30 and 31 kDa by ELISA and confirmed by Western blot in the CSF and indeterminate in the serum, probably due to late seroconversion, in addition to detectable PCR for *A. cantonensis* in the CSF.

Meningitis caused by *A. cantonensis* is treated by reducing the inflammatory response and headache for which the use of corticosteroid therapy is indicated, generally in the form of prednisolone 60 mg/kg/day for 14 days^9^. Among anti-helmintics, the use of albendazole 15 mg/kg twice a day for 14 days is recommended^14^. Some studies have administered mebendazole 10 mg/kg/day, but the use of these medications triggered a severe inflammatory response due to the death of the parasites, which is why it is not recommended^9,14^. Relief lumbar punctures to reduce headache has proven benefit^14^.

Regarding prophylaxis, promoting both personal hygiene and the adequate washing and cooking of food for consumption, especially vegetables, raw shrimp or seafood, is necessary^9^.

## CONCLUSION

Eosinophilic meningitis caused by *Angiostrongylus cantonensis* is a rare and difficult-to-diagnose condition, requiring increased awareness and inclusion of neuroangiostrongyliasis in the differential diagnosis of eosinophilic meningitis. As shown in this report, false-positive reactions with immunological tests for *N. meningitidis* can hinder the best patient management.

## Data Availability

The complete anonymized dataset supporting the findings of this study is included within the article itself.
